# Care negotiation between people living alone with dementia in the United States and non‐kin caregivers

**DOI:** 10.1002/alz.087719

**Published:** 2025-01-09

**Authors:** Jane Lowers, Colby Smith, Kaitlyn Brus, Molly Perkins, Kenneth Hepburn

**Affiliations:** ^1^ Emory University, Atlanta, GA USA; ^2^ Emory University Nell Hodgson Woodruff School of Nursing, Atlanta, GA USA

## Abstract

**Background:**

One in five people with cognitive impairment or dementia lacks family caregivers. People aging alone receive care from non‐family sources, including friends and neighbors, but the dynamics of these relationships are unexplored.

**Method:**

Thematic analysis of qualitative interviews with 15 people with cognitive impairment or early dementia without close family and 14 non‐family caregivers

**Result:**

We identified five themes that shape non‐family dementia caregiving dynamics: 1) establishing a relationship of trust, 2) personal motivations for seeking/providing care, 3) negotiating caregiving etiquette, 4) establishing caregiving boundaries, 5) the role of distant family.

**Conclusion:**

Dementia caregiving by non‐family involves ongoing interpersonal negotiation of trust, roles, and boundaries.

